# Hajj Pilgrims’ Knowledge about Acute Respiratory Infections

**DOI:** 10.3201/eid1511.090201

**Published:** 2009-11

**Authors:** Philippe Gautret, Georges Soula, Philippe Parola, Philippe Brouqui

**Affiliations:** Hôpital Nord, Assistance Publique-Hôpitaux de Marseille, Marseille, France

**Keywords:** Influenza, viruses, Hajj, respiratory tract infections, preventive measures, masks, letter

**To the Editor:** Hajj pilgrimage is a yearly event in which >2 million Muslims from around the world gather in Mecca, Saudi Arabia. Such high density of crowding presents a risk for local outbreaks and for worldwide spread of infectious agents. Acute respiratory infection (ARI) is the leading cause of admission to Saudi hospitals during the Hajj (*1*). In Marseille, France, after administration of systematic questionnaires, we recorded attack rates of ARI up to 60% in cohorts of returned Hajj pilgrims in 2006 (*2*). This potential risk is of particular concern because of the influenza A pandemic (H1N1) 2009 virus (*3*). ARI transmission can be efficiently reduced by simple, low-cost physical measures, including use of face masks and hand hygiene. Awareness and acceptability of these measures among pilgrims, however, are limited (*4*).

We conducted a knowledge, attitudes, and practices survey that addressed these issues among Hajj pilgrims departing from Marseille during October and November 2008, several months before the outbreak of pandemic (H1N1) 2009 virus. A total of 528 persons (290 males, 238 females) who attended a pre-Hajj meningococcal vaccination campaign were invited to participate in a face-to-face interview during which they completed our questionnaire. We achieved a 100% response rate. Mean age of participants was 61 years (range 18–94 years). Most pilgrims were born in North Africa (92%), had education above a primary certificate (81%), were unemployed (56% of persons <65 years of age), and were traveling to Saudi Arabia for the first time (78%). Ten percent had chronic pulmonary disease.

We assessed knowledge of ARI using 18 questions about symptoms and sources of contamination. Knowledge questions were scored 1 for the correct answer and 0 for incorrect or “don’t know” answers. Overall, the score of true responses was only 26% (interquartile range [IQR] 21%–37%). Scores were higher for respondents <65 years of age (32% [IQR 21%–42%] vs. 26% [IQR 16%–32%], p<0.00001 by Kruskal-Wallis test). Scores were also higher for female pilgrims (32% [IQR 21%–37%] vs. 26% [IQR 21%–37%, p = 0.01). No other demographic or health factor had significant influence.

Respondents believed the following were sources of contamination for ARI: sneeze and cough products (58.1%), dirty hands (43.9%), contact with ill persons (40.5%), saliva (17.2%), promiscuity (17.0%), food (12.1%), drink (9.1%), air conditioning (3.4%), and contact with animals (0.4%); 16.7% had no knowledge about ARI sources. When asked about their perceived risk of acquiring ARI during the pilgrimage and contaminating their relatives on returning home, 26% of respondents perceived no or little risk, 20% perceived some risk, and 37% perceived important risk; 17% did not know. Surveyed pilgrims knew the following were symptoms of ARI: cough (64.4% of respondents), dyspnea (45.1%), fatigue (33.3%); expectoration (21.0%), fever (15.2%), rhinitis (8.7%), nasal obstruction (4.0%), and headache and sneeze (3.8% each); 14.4% of pilgrims surveyed did not know any ARI symptoms. Less than 50% of respondents were aware of social distancing, curative treatment, and use of a face mask as precautions to reduce the spread of ARI agents (Table). However, when informed about the effectiveness of those prevention measures, most pilgrims were willing to wear a mask (92%), frequently wash their hands (98%), use hand disinfectants (89%), and use disposable handkerchiefs (97%) (Table).

**Table:** Knowledge and acceptability of precautions against acute respiratory tract infections in French Hajj pilgrims, October–November 2008

Precaution	Knowledge about prevention measure		Acceptability of prevention measure, %
	Use for self-protection, %	Use for community protection, %	
Use of face mask	41.3	24.6	91.7
Hand washing	9.8	6.4	92.8
Use of hand disinfectant	2.8	1.9	98.1
Use of disposable handkerchief	–	1.1	96.8
Social distancing	48.7	57.4	62.5
Contact avoidance	47.0	54.7	62.1
Preventive treatment	14.6	–	–
Preventive vaccination	16.9	–	94.7
Curative treatment	–	46.2	97.7
No idea	11.2	7.8	–

Saudi health authorities recommend use of surgical face masks (*5*); however, data conflict about the protective effect of such masks during the pilgrimage (*5*,*6*). Use of face masks varies according to the origin of Hajj pilgrims; in 1 study, only 15% of pilgrims from the Middle East, 17% from Europe and the United States, and 45% from Southeast Asia used a mask (*4*). Promotion and distribution of free masks increased their use from 34% to 81% in another cohort of Saudi pilgrims (*6*). National Health Service for England does not advise the use of masks, considering compliance with this advice unlikely because many Muslims believe that covering the face during the Hajj is prohibited and because masks need to be of high quality and changed at least every 6 hours to remain effective (*7*). Recent studies demonstrated that surgical and N95 masks were equally effective in preventing spread of PCR-detectable influenza virus when used by infected patients. These masks also were potentially effective at preventing respiratory virus acquisition by household contacts of infected persons when worn by healthy persons. However, effectiveness depended largely on adherence to mask use (*8*,*9*).

Maintenance of good hand hygiene is also effective in reducing spread of respiratory infection. The World Muslim League has issued a *fatwa* allowing use of alcohol-based hand-rubs on skin as a disinfectant (*10*).

The demonstration of high acceptability of simple physical measures to prevent ARI encourages the education of pilgrims during the pretravel encounter. The results also support conclusion that masks, hand-rubs, and disposable handkerchiefs should be provided to pilgrims, along with strong advice about the risk for ARI, to increase adherence to prevention measures.
